# Descriptive sensory analysis and consumer preferences of bean sauces

**DOI:** 10.1002/fsn3.1721

**Published:** 2020-07-16

**Authors:** Rachel Byarugaba, Agnes Nabubuya, John Muyonga

**Affiliations:** ^1^ Department of Food Technology and Nutrition School of Food Technology, Nutrition and Bioengineering College of Agricultural and Environmental Sciences Makerere University Kampala Uganda

**Keywords:** consumer preference, processed beans, quantitative descriptive analysis, sensory acceptability

## Abstract

Sensory acceptability of value‐added bean products is a critical determinant of their consumption. This study determined the factors influencing consumer preference of processed beans. Dry common beans (*Phaseolus vulgaris* L) processed by boiling, roasting, and extrusion were milled into flour and used to make bean sauces. The sauces were evaluated by 10 panelists using quantitative descriptive analysis and ranked by 120 consumers for preference. The factors influencing consumer preference were computed by a partial least squares regression model. The results showed that sauces were more distinguishable by appearance, taste, and mouth‐feel than by aroma, flavor, and after‐taste. Sauces that were brown in color, with burnt aroma and burnt after‐taste were preferred to those that were described as lumpy with mashed potato flavor. Oven roasted beans and boiled beans were preferred to traditionally roasted, extruded, and unprocessed beans. Preference was significantly (*p* < .05) positively influenced by brown color and negatively by lumpiness which were both sensory descriptors of the appearance of sauce. Appearance is therefore the leading influencer of consumer preference in bean sauces and should be prioritized by product developers over other sensory attributes in development of similar products for wider acceptance and utilization of common beans.

## Graphical abstract

Different processing methods yielded bean sauces more distinguishable by appearance, taste, and mouth‐feel than by aroma, flavor, and after‐taste. Sauces that were brown in color, with burnt aroma and burnt after‐taste were preferred to those that were described as lumpy with mashed potato flavor. Oven roasted beans and boiled beans were preferred to traditionally roasted, extruded, and unprocessed beans.

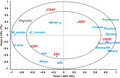

## 1 INTRODUCTION

Common beans (*Phaseolus vulgaris* L) are a good source of protein (de Almeida Costa, da Silva Queiroz‐Monici, Reis, & de Oliveira, 2006; Siddiq, Butt, & Sultan, 2011), dietary fibre, starch (Osorio‐Díaz et al., 2003), minerals, vitamins (Kutos, Golob, Kac, & Plestenjak, 2003), and beneficial nutraceuticals such as polyphenols (Wu et al., 2004). Consumption of dry beans has the potential to impart a variety of health benefits, including reduction of the risk for developing diabetes mellitus (Thompson, Winham, & Hutchins, 2012), obesity (Anderson & Major, 2002), cardiovascular diseases (Anderson, Smith, & Washnock, 1999), and colon diseases (Tharanathan & Mahadevamma, 2003). In developing countries where protein energy malnutrition is still rampant, dry beans are particularly useful for promoting nutrition security (Venter & van Eyssen, 2001). With increasing urbanization, consumer preferences are shifting in favor of convenience foods and commodities, which require reduced food preparation time (Siddiq & Uebersax, 2012). However, there is limited availability of novel products and ingredients from beans that are convenient and potentially useful in traditional recipes and cuisine (Uebersax, 2006). The implication of urbanization coupled with awareness on healthy eating and a higher number of the working class is that, the demand for processed beans and bean products is anticipated to increase as people seek to save time used in food preparation (Siddiq & Uebersax, 2012). Processing dry beans into value‐added products has potential to increase their utilization since it permits for incorporation of beans into different food systems (McDermott & Wyatt, 2017; Uebersax, 2006).

Commercial bean flours are generally produced by means of extrusion, or by a process involving soaking, blanching and steam cooking followed by drying and milling (Subuola, Widodo, & Kehinde, 2012; Szczygiel, Harte, Strasburg, & Cho, 2017). These processes eliminate flatulence‐causing oligosaccharides and anti‐nutrients (de Almeida Costa et al., 2006; Kelkar et al., 2012; Siddiq, Kelkar, Harte, Dolan, & Nyombaire, 2013). However, the commercial processes use a significant amount of water or/and energy. In contrast, in parts of Uganda, bean flour is made traditionally by roasting dry beans followed by milling. To be able to mill whole beans without soaking and dehulling and maintain sensory acceptability in developed products would be a more appealing option for bean flour producers from an economical and environmental stand point (Szczygiel et al., 2017).

Processing methods appear to be a major determinant of the sensory properties of dry beans. Siddiq et al. (2013) used low temperature extrusion and steam cooking to produce navy and pinto bean powders. Sensory tests showed that cookies made from powder from extruded beans were more acceptable than those from powder from steam cooked beans. Nyombaire, Siddiq, and Dolan (2011) used bean extrudates to produce porridge and reported that a consumer panel could not detect beany flavor. Simons (2013) found that extruded 100% bean snacks were able to exceed the minimum flavor value for acceptability, but also reported that many panelists noted an undesirable “beany” flavor. Han, Janz, and Gerlat (2010) reported that crackers developed from unprocessed navy bean were not highly acceptable due to a strong “beany” flavor. He also noted that flavor is likely to be the largest challenge in producing bean‐based products.

Bean flour is susceptible to development of flavor by degradation or modification of phytochemicals and odor‐active chemicals inherently present in the beans (Heng et al., 2006). These could range from furans from Maillard reaction products to aliphatic aldehydes from lipid degradation (Simons, 2013). Because bean flour is usually precooked, many off‐flavor compounds are reduced, removed or altered during processing and the remaining cooked beany flavors become the primary concern for sensory issues related to flavor (Shi et al., 2004). Mkanda, Minnaar, and De Kock (2007) reported that factors that contributed to dislike of boiled dry beans were bitter taste and soapy and metallic after‐tastes. Simons (2013) proposed that extruded bean powders may have superior flavor properties compared with those produced by steam cooking because of the lipids that are less available to be oxidized and produce off‐flavors.

Preference mapping techniques are well known for their use in sensory analysis to relate descriptive sensory data and consumer preference data with the purpose of identifying the drivers of liking (Lawless & Heymann, 2010). Van Kleef, van Trijp, and Luning (2006) reported that external preference mapping is more useful in understanding the differences in preferences from the product point of view, while internal preference mapping is more appropriate to understand consumers' preferences. While descriptive sensory evaluation is used to determine the nature of the differences between products (Stone & Sidel, 2004), determining consumer preferences or acceptability of the products is potentially useful in directing the production and marketing industries (Booth, 1998) on product development for commercial success. Roasted legumes have been reported to be characterized by unique flavors which can increase their sensory appeal (Subuola et al., 2012). However, there is little information in literature on the sensory attributes of products from roasted bean flour and how these relate to consumer preferences. The objectives of this study were to describe the sensory properties of sauces made from processed bean flour and to establish the sensory properties that influence preference of the sauces.

## 2 MATERIALS AND METHODS

### 2.1 Sample collection and preparation

Dry red common beans (NABE 15) locally (in Uganda) known as *Kanyebwa* which are commonly grown in all bean growing regions within Uganda were purchased from Owino market, in Kampala. According to the beans value chain assessment survey done in Uganda, *Kanyebwa* was considered fairly priced and ranked high in terms of consumer preference because of attributes such as short cooking time, thick soup, swelling characteristics, good taste, and familiarity (Kilimo Trust, 2012).

Beans were purposively sampled from lots that had been verified to be freshly dried by means of observation of color, smell, and absence of weevils. Three 50 kg sacks of freshly dried beans were obtained from a supplier. After mixing the contents of a sack by means of diagonal quartering several times, 15 kg of beans were weighed from each sack. Replication was achieved by collecting beans from an additional two suppliers from the same market using the same approach. The beans were sorted by hand to remove extraneous materials like dry leaves, stones, and dirt. The beans were washed twice with portable water to remove dust and solar dried at 46–54°C in a dome solar dryer for 8 hr to remove any moisture acquired during washing.

### 2.2 Production of flours

The beans were divided into portions to be processed using different techniques. A control was left unprocessed and milled into bean flour. The flour was packaged into an air tight plastic container and stored at room temperature pending further analysis.

#### 2.2.1 Production of flour from oven roasted beans

A portion of the beans (1 kg) was roasted in an oven (Infrared food oven GL‐2A, Guangzhou Itop Kitchen Equipment Co, Ltd.) at 185.9°C for 7.5 min on stainless steel trays (0.75 m by 0.5 m) while stirring periodically with a wooden spoon to ensure uniform heat distribution. The aforementioned temperature and time combination had been established from trials as being optimal for roasting dry beans. After cooling, the roasted beans were ground to fine flour using a mill (Wonder mill) and sieved through 425 microns mesh size. The flour was packaged into an air tight plastic container and stored at room temperature pending further analysis.

#### 2.2.2 Production of bean flour by pan roasting

This was done by roasting the beans in a metallic saucepan on a charcoal stove for 7 min while stirring periodically with a wooden spoon to ensure uniform heat distribution. After cooling, the roasted beans were milled and stored as described in Section 2.2.1.

#### 2.2.3 Production of flour from boiled beans

Boiling was included among the processing methods because it is commonly used to cook beans for home consumption. Dry beans were boiled in a covered saucepan with water (1:3 w/v) for 69 min (Nyakuni et al., 2008). After draining off excess water, the boiled beans were dried in a cabinet dryer at 55°C for 12 hr. The dry beans were milled and stored as described in Section 2.2.1.

#### 2.2.4 Production of flour from extruded beans

A portion of the beans (50 kg) was extruded using a twin‐screw extruder model DP‐70‐III (Jinan Eagle Food Machinery Co., Ltd.) with three heating sections; the first set at 60°C, the second at 120°C and the last one at 140°C. The extruder filler was set at a speed of 30 Hz (900 rpm), the screw at 35 Hz (2100 rpm), and the cutters at a 30 Hz (900 rpm). The diameter was 4 mm, and the beans were extruded at 15% moisture content. The extruded beans were milled and stored as described in Section 2.2.1.

### 2.3 Preparation of bean sauces

Bean flours produced using the different processing methods plus the control were used to prepare sauces. A recipe for tomato‐bean sauce (Natabirwa, Katende, & Lung'aho, 2014) where bean grains were replaced with bean flour was used. The ingredients used included 13 g of onions or ¼ of an onion; 12 g or ¼ a green pepper; 35 g or ¼ medium sized tomato, which were all chopped and shallow fried in 5 ml of vegetable cooking oil and about 1 g of curry powder and 5 g of salt added to the mixture. The bean flour (100 g) was then stirred into the mixture after which 1,200 ml of water was added gradually with constant stirring to avoid lumping. The sauce was cooked for 10 min.

### 2.4 Determination of viscosity

A Rapid Visco‐Analyzer (RVA 4500; Newport Scientific Pty. Ltd.) was used to determine the final viscosity of bean sauces at 50°C, which was also the approximate temperature at which sauces were served. To simulate cooking, 3.5 g of flour, 1.5 g of a mixture of pureed onion, tomato, green pepper, curry powder, salt, and oil were added to 25 ml of water in a canister and subjected to standard profile 1. This involved 1 min of mixing and warming up at 50°C; 3.7 min of heating at 12°C/min up to 95°C; 2.5 min of holding at 95°C; 3.8 min of cooling down to 50°C at 12°C/min and 2 min of holding at 50°C. A constant paddle rotational speed (160 rpm) was used throughout the entire analysis, except for rapid stirring at 960 rpm for the first 10 s to disperse the sample. Analyses were done in triplicate and results for final viscosity at 50°C were recorded.

### 2.5 Descriptive sensory analysis

#### 2.5.1 Panel selection and training

Analytical methods for differentiation were used in the selection of panelists, and descriptive quantitative analysis (Meilgaard, Carr, & Civille, 1999) for the evaluation of bean sauces. Ten people all students and staff in the Department of Food Technology and Nutrition at Makerere University were recruited. Participation as panelists in descriptive sensory analysis of the bean sauces was based on frequency of bean consumption (at least twice per week), sensory acuity determined by basic taste recognition test and triangle test (Meilgaard, Carr, & Civille, 2006), interest and availability. Seven men and three women in the age range of 20–32 years were selected. Consensus training as explained by Lawless and Heymann (1999) was conducted. Panelists were exposed to bean sauces to be tested in the descriptive analysis sessions. Through consensus, panelists generated terms (descriptors) and definitions to describe the sensory differences perceived among the samples. Panelists also decided on words to anchor the descriptive terms and some reference standards to be used (Table 1). Fourteen sensory descriptors/attributes describing appearance, aroma, flavor, taste, mouth‐feel, and after‐taste were generated (Table 1). Two training sessions per week were held for 8 weeks and each session lasted 2 hr. Before the actual evaluation, trial evaluations were performed and the panelists' performance was checked using Panel Analysis tool in XLSTAT (version 2012. 10.7.01 Addinsoft) to enable decisions on panelists' reproducibility.

**TABLE 1:** Descriptors, their definitions, and references used to explain sensory perceptions of bean sauces

Descriptor	Code	Definition	Reference
Appearance			
Brown	Brown	Intensity of brown color of the bean sauces	Color wheel
Lumpiness	Lump	The amount of ball like structures in the sauce regardless of size	Porridge from extruded millet soya (15% w/v) = 15
Aroma			
Beany	Beany	The aroma associated with cooked dry beans	Boiled dry beans
Burnt	Burnt	The aroma similar to burnt beans	Burnt boiled beans
Flavor			
Mashed potato	M_potato	The flavor associated with mashed boiled potato	Freshly boiled, mashed potato
Cooked tomato	C_tomato	The intensity of the flavor associated with cooked tomato	Steamed whole tomato
Millet porridge	Millet_p	The intensity of the flavor associated with un‐malted millet porridge	Un‐malted millet porridge
Fried bean stew	Stew	The intensity of the flavor associated with freshly fried pre‐boiled dry beans	Soup from fried, pre‐boiled whole beans
Taste			
Saltiness	Salt	The basic salt taste associated with sodium chloride (table salt)	NaCl; 0.2% = 2, 1.5% = 15 intensity
Mouth‐feel			
Coarseness	Coarse	The grittiness or graininess of sauce caused by small particles that could be perceived in the mouth	Porridge from malted sorghum (35% w/v) = 10
Particulate	Particul	The degree to which sauce leaves particles on the tongue after swallowing	Porridge from whole maize flour (8% w/v) = 10
After‐taste			
Beany after‐taste	Beanyaft	The intensity of lingering taste similar to freshly cooked dry beans	Broth from boiled dry beans
Burnt after‐taste	Burntaft	The intensity of lingering taste similar to burnt beans	Broth from burnt boiled beans
Saltiness after‐taste	SA	The intensity of lingering basic salt taste associated with sodium chloride (table salt)	NaCl; 0.02% = 2, 0.15% = 15 intensity

#### 2.5.2 Sample presentation

Five sauces were prepared. About 50 g of each sauce was served in a plastic bowl labeled with a three‐digit random number. The temperature of the samples at the time of evaluation was about 50°C. The samples were presented in random order to the panelists for evaluation, and each product was assessed alone. Panelists were asked to eat at least one spoonful of each sample.

#### 2.5.3 Descriptive analysis procedure

The 10 panelists rated attribute intensities of the five bean sauces on a questionnaire using a continuous, unstructured 15 cm line scale (Meilgaard et al., 2006) anchored at the ends by low and very pronounced for most attributes. Exceptions included: coarseness which was anchored by smooth and particulate; lumpiness which was anchored by none and many, and brown which was anchored by light and dark. Each panelist evaluated the products individually. The evaluation was conducted in well‐ventilated sensory booths fitted with fluorescent lights. Panelists evaluated attributes starting from aroma, followed by flavor, and subsequently any attribute of their choice as clearly indicated in the instructions on the questionnaire. Between the samples, panelists rinsed their mouths with water. Products were evaluated in three sessions on 3 consecutive days. The sessions acted as replicates.

### 2.6 Consumer preference test

Students and staff members from the Department of Food Technology and Nutrition at Makerere University campus were invited to participate in the test. Prior to analysis, consumers were questioned about the frequency of their bean consumption and only consumers who consumed beans at least once per week were invited to participate in the preference test. Consumers were presented with questionnaires on paper and rated their preference for each of the five bean sauces on a nine‐point preference scale (Balthazar et al., 2018) where 1 was “dislike extremely” and 9 “like extremely.” Consumers were asked to select which bean sauce they would buy and mention reasons for their choice in addition to making any other comments about the bean sauces. Testing conditions were similar to those used for descriptive analysis as described in Section 2.5.3. All products were served at each session following a completely randomized design (Lawless & Heymann, 2010). One hundred and twenty consumers aged 20–44 years participated in the test.

### 2.7 Statistical analysis

During training, the whole panel was calibrated by obtaining the mean rating. Panelists whose rating was not within 10% of the mean rating were asked to re‐evaluate the samples and references and adjust their ratings until a consensus was reached. Panelists' reproducibility was determined using analysis of variance (ANOVA) at *p* = .05 in the Panel Analysis tool in XLSTAT (version 2012. 10.7.01 Addinsoft). Panelists whose ratings were not reproducible were assisted to improve performance. At the end of the descriptive analysis, Panel Analysis tool was used to assess panelists' consensus, the discrimination ability of the descriptors and whether there were assessor and session effects (Raithatha & Rogers, 2018). Only descriptors which significantly (*p* < .05) discriminated between different bean sauces were included in subsequent analysis. Mean intensity scores of the descriptors and mean viscosities for the different bean sauces were compared using ANOVA. Significant differences between the sauces were assessed by Tukey's post hoc test. To understand how the sensory descriptors and viscosity characterized the bean sauces, principal component analysis (PCA) was performed. The two‐way matrix data set used consisted of five rows (bean sauces) and 20 columns (five as dummy variables of the bean sauces, 14 for sensory descriptors and one for viscosity).

Internal preference mapping was used to analyze consumer preference data (Meilgaard et al., 2006). It was carried out using PCA of the individual consumer preference scores. The two‐way matrix data set used consisted of five rows (bean sauces) and 125 columns (five as dummy variables of the bean sauces and 120 as individual consumer preference scores). The result was a plot in which consumers were represented as individual variables depicting the differences in preferences for each of the bean sauce samples. Prior to PCA, the data were standardized (1/*SD*). To segment consumers into subgroups sharing common preference patterns, hierarchical cluster analysis (HCA) with squared Euclidian distances and Ward's method was carried out on the consumer preference scores (Carbonell, Izquierdo, Carbonell, & Costell, 2008). ANOVA was used for identification of differences in preference among the clusters. Least significance difference (LSD) post hoc test for multiple comparisons was used to identify where differences existed since the clusters had different sizes.

Finally, two partial least squares regression models (PLSR) were built for studying the relationship between sensory descriptors and consumer preference (Hasted, 2018). The PLSR1 (single response) was carried out by regressing the average preference score for all consumers onto the sensory descriptors in order to identify the relevant sensory attributes that influenced the preference of the consumers. Sensory descriptors and viscosities of the bean sauces were used as explanatory variables (X matrix) while means of overall consumer preference for each bean sauce were used as the response variable (Y matrix). The two‐way matrix data set used consisted of five rows (bean sauces) and 21 columns (five as dummy variables of the bean sauces, 14 for sensory descriptors, one for viscosity and one for overall consumer preference score for each bean sauce). The PLSR2 (multiple responses) was done by regressing the sensory characteristics and viscosity readings onto clusters obtained from the hierarchical clustering of the preference data in order to understand attributes influencing preference of consumers in these clusters. Sensory descriptors and viscosities of the bean sauces were used as explanatory variables (X matrix) while means of overall consumer preference for each bean sauce in the different clusters were used as the response variable (Y matrix). The two‐way matrix data set used consisted of five rows (bean sauces) and 23 columns (five as dummy variables of the bean sauces, 14 for sensory descriptors, one for viscosity, and three for the average consumer preference score for each bean sauce in each of three consumer clusters). PCA and PLSR analysis commonly require pretreatment or pre‐processing of the data (Skov, Honoré, Jensen, Næs, & Engelsen, 2014), such as “normalization” and scaling, to remove systematic bias from the data sets, but with minimal influence on the quality of information (Nunes, Alvarenga, de Souza Sant'Ana, Santos, & Granato, 2015). Data used in PCA and PLSR were centered to remove bias associated with the magnitude of variable means, full cross‐validated to test predictive ability and standardized to allow comparison. ANOVA and cluster analysis were performed using SPSS (version 22.0.; SPSS Inc) while PCA and PLSR were performed in Unscrambler X 10.5 (CAMO Software) and XLSTAT (version 2012. 10.7.01 Addinsoft).

## 3 RESULTS AND DISCUSSION

### 3.1 Sensory description and viscosity of sauces from beans

The ANOVA results showed that all fourteen sensory descriptors were significantly discriminating (*p* < .05) among the bean sauce samples and were retained for use in subsequent analysis. There were no session effects (*p* > .05) on the results for the bean samples. Panelists distinguished between the bean sauces more by their appearance, taste, and mouth‐feel than by the aroma, flavor, and after‐taste descriptors (Table 2). Sauce from unprocessed flour had higher viscosity than sauces from the processed flours except for sauce from traditionally roasted bean flour (TRBF). A similar trend has been reported for viscosity of flours from raw, extruded, and malted and roasted common beans (Nkundabombi, Nakimbugwe, & Muyonga, 2016) as well as tempered and heat dried navy and pinto beans (Anton, Ross, Beta, Fulcher, & Arntfield, 2008). Boiling, extrusion and oven roasting contributed to modification of starch leading to the reduced ability of flour from processed beans to form a thick paste after gelatinization compared with the flour from unprocessed beans which had native starch. The increase in heat treatment used in oven roasting compared with traditional roasting is likely to have caused a high degree of starch dextrinization (Sacchetti, Pinnavaia, Guidolin, & Dalla Rosa, 2004). This, coupled with possible high level of retrogradation of the bean starch (Sandhu & Singh, 2007) resulted in greater reduction of final viscosity.

**TABLE 2:** Mean panel scores and standard deviations for descriptive sensory attributes and viscosity of the bean sauces

Sensory descriptor	UBF	TRBF	BBF	EBF	ORBF	*p*‐value
Appearance						
Brown	1.62 ± 0.21^a^	4.46 ± 0.19^b^	8.68 ± 0.19^d^	5.60 ± 0.19^c^	13.75 ± 0.28^e^	<.0001
Lumpiness	11.70 ± 0.17^e^	7.60 ± 0.29^c^	1.86 ± 0.18^b^	8.69 ± 0.16^d^	0.87 ± 0.17^a^	<.0001
Aroma						
Beany	6.08 ± 0.39^a^	5.87 ± 0.48^a^	6.87 ± 0.38^b^	8.47 ± 0.20^c^	11.35 ± 0.31^d^	<.0001
Burnt	0.46 ± 0.09^a^	1.50 ± 0.14^c^	0.64 ± 0.14^b^	2.24 ± 0.28^d^	11.67 ± 0.26^e^	<.0001
Flavor						
Mashed potato	4.51 ± 0.20^e^	2.79 ± 0.15^d^	1.53 ± 0.14^b^	1.70 ± 0.11^c^	0.41 ± 0.12^a^	<.0001
Cooked tomato	5.54 ± 0.21^b^	6.41 ± 0.21^c^	6.55 ± 0.15^d^	7.52 ± 0.18^e^	2.54 ± 0.14^a^	<.0001
Millet porridge	2.55 ± 0.15^b^	2.57 ± 0.21^b^	5.08 ± 0.28^c^	2.55 ± 0.21^b^	0.52 ± 0.10^a^	<.0001
Fried bean stew	9.53 ± 0.20^d^	7.40 ± 0.17^b^	5.50 ± 0.15^a^	9.61 ± 0.16^d^	8.56 ± 0.18^c^	<.0001
Taste						
Saltiness	7.88 ± 0.33^c^	5.80 ± 0.31^b^	2.52 ± 0.18^a^	9.64 ± 0.27^e^	8.56 ± 0.23^d^	<.0001
Mouth‐feel						
Coarseness	2.56 ± 0.13^a^	3.63 ± 0.22^b^	7.53 ± 0.15^e^	4.41 ± 0.15^c^	5.71 ± 0.21^d^	<.0001
Particulate	2.30 ± 0.18^a^	3.35 ± 0.16^b^	7.33 ± 0.22^e^	4.36 ± 0.18^c^	5.57 ± 0.19^d^	<.0001
After‐taste						
Beany	3.72 ± 0.21^b^	3.53 ± 0.19^a^	4.60 ± 0.23^c^	5.63 ± 0.24^d^	9.58 ± 0.14^e^	<.0001
Burnt	0.49 ± 0.11^a^	1.00 ± 0.20^c^	0.69 ± 0.11^b^	1.46 ± 0.13^d^	7.55 ± 0.22^e^	<.0001
Saltiness	6.62 ± 0.13^c^	5.79 ± 0.28^b^	2.59 ± 014^a^	6.62 ± 0.13^c^	5.72 ± 0.23^b^	<.0001
Viscosity (RVU)	168.11 ± 4.43^c^	194.75 ± 5.34^d^	14.11 ± 0.05^a^	26.03 ± 2.60^b^	19.36 ± 0.38^a^	.000

Note

Values are means of triplicate determinations. Values are presented as mean ± *SD*. Means having different superscripts within the same row are significantly different at *p* ≤ .05.

Abbreviations: BBF, boiled bean flour; EBF, extruded bean flour; ORBF, oven roasted bean flour; TRBF, traditionally roasted bean flour; UBF, unprocessed bean flour.

**FIGURE 1:**
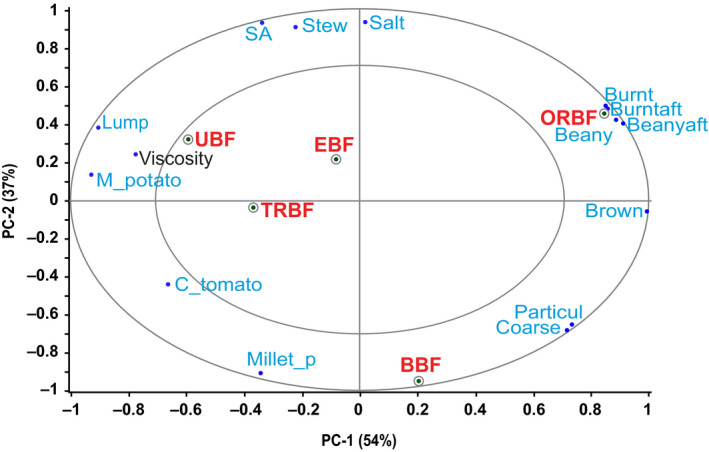
PCA results showing description of the bean sauces by sensory attributes and viscosity. BBF, boiled bean flour; Beanyaft, beany after‐taste; Burntaft, burnt after‐taste; C_tomato, cooked tomato; Coarse, coarseness; EBF, extruded bean flour; Lump, lumpiness; M_potato, mashed potato; Millet_p, millet porridge; ORBF, oven roasted bean flour; Particul, particulate; SA, salt after‐taste; Stew, fried bean stew; TRBF, traditionally roasted bean flour; UBF, unprocessed bean flour

Principal component analysis results showed that the first two principal components (PCs) explained 91% of the sensory data variation. Bean sauces from oven roasted bean flour (ORBF) and boiled bean flour (BBF) were clearly distinguished from the other three bean sauces (Figure 1). PC1 which explained 54% of the variance in the sensory data, predominantly described the sauce from ORBF while PC2, accounting for 37% of the variation in the sensory data mainly described the sauce from BBF. The PCA results also showed an anti‐correlation between the sauce from traditionally roasted bean flour (TRBF) and ORBF sauce.

Oven roasted bean flour (*r* = .85, PC1) was characterized as being highly expressive of coarseness, particulate mouth‐feel, brown appearance, beany, and burnt aroma, beany and burnt after‐tastes. On the other hand, the same sample had low lumpiness, low mashed potato flavor, and low viscosity. Burnt aroma was reported by Nyembwe, Minnaar, Duodu, and de Kock (2015) in pastes from roasted marama beans. According to Ma, Boye, Azarnia, and Simpson (2016), volatile compounds such as pyrazines and alkylated pyrazines produced during roasting may play an important role in masking the beany flavor associated with the presence of aldehydes, alcohols, and sulfur compounds in raw pulses. Yoon (1996) and Kim, Yoon, and Rhee (2000) also reported that pyrazines, most important being 2,5 dimethylpyrazine and 2‐methylpyrazine may be responsible for burnt aromas in roasted sesame and perilla seed oils, respectively. These compounds probably contributed to the more intense burnt aroma in sauces made from ORBF whose beans were roasted at a higher temperature than those of TRBF. A study by Tomlins, Rukuni, Mutungamiri, Mandeya, and Swetman (2007) on sensory attributes of peanut butter, demonstrated that increased roasting was associated with sensory attributes such as brown color. High processing temperatures enhance the Maillard reaction, whose final products are melanoidins which are responsible for brown color development (Delgado‐Andrade, Rufián‐Henares, & Morales, 2005). Szczygiel et al. (2017) reported beany aroma in extruded and commercial navy bean powders whose intensity was contributed by the volatile compounds hexanal, 3,5‐octadien‐2‐one, and benzaldehyde (Gallegos‐Infante et al., 2010; Han et al., 2010) which are considered the authentic volatile markers for dry beans (Oomah, Liang, & Balasubramanian, 2007).

The sauce from BBF (*r* = −.95, PC2) was characterized by the high expression of coarseness, particulate mouth‐feel and millet porridge flavor but had low expression of fried bean stew flavor, low saltiness and saltiness after‐taste as well as low viscosity. Sauce from unprocessed bean flour (UBF; *r* = −.59, PC1) was characterized as being lumpy with high mashed potato flavor and high viscosity. Mishra, Tripathi, Gupta, and Variyar (2017) reported boiled potato aroma in cooked kidney beans. Mashed potato flavor can be attributed to the major aroma component of boiled potatoes methional (Maga, 1994; Mutti & Grosch, 1999; Ulrich, Hoberg, Neugebauer, Tiemann, & Darsow, 2000). Methional is a major aroma compound that is formed by the Strecker degradation reaction from conversion of the amino acid methionine. Methional can be further oxidized into a variety of potent aroma compounds such as methanethiol, and dimethyl sulfide. Lovegren, Fisher, Legendre, and Schuller (1979) reported methanethiol, methylated sulfides, and 2‐methylthiophene in dried kidney beans. Mishra et al. (2017) reported methional, methanethiol, diethyl sulfide, dimethyl disulfide, and dimethyl trisulfide, as contributing to aroma in cooked kidney beans. Unlike the three previous samples, bean sauces from TRBF and extruded bean flour (EBF) were not predominantly characterized by any sensory descriptor. It appeared that panelists were more able to differentiate sauces from ORBF, BBF, and UBF than those from TRBF and EBF. This was based on the fact that values for TRBF and EBF loaded averagely on both PC1 and PC2.

Significant correlations were observed among the sensory descriptors (Table 3). Judging from the strong negative correlations observed among the appearance attributes, the darker the brown color of the bean sauce, the less likely it was to be lumpy or have mashed potato flavor. It appeared that fried bean stew flavor, saltiness, and saltiness after‐taste enhanced each other as seen from their strong positive correlations. Although the same amount of salt was added to all the samples, its perception varied. Salt content alone does not sufficiently predict perceived saltiness intensity as synergistic interactions of salt and other flavor compounds also affect saltiness perception (Kim, Hong, Song, Shin, & Kim, 2010). Beany aroma in bean sauces strongly guaranteed presence of burnt aroma, and the same was true for coarseness and being particulate.

**TABLE 3:** Pearson (*n*) correlations between sensory descriptors and viscosity characterizing the bean sauces

Variables	Brown	Lump‐iness	Beany	Burnt	Mashed potato	Cooked tomato	Millet porridge	Fried bean stew	Salt‐iness	Coarse‐ness	Particulate	Beany after‐taste	Burnt after‐taste	Salt‐iness after‐taste	Viscosity
Brown	**1**														
Lumpiness	**−0.93**	**1**													
Beany	0.85	−0.61	**1**												
Burnt	0.84	−0.61	**0.93**	**1**											
Mashed potato	**−0.92**	0.86	−0.80	−0.69	**1**										
Cooked tomato	−0.66	0.50	−0.69	−0.87	0.36	**1**									
Millet porridge	−0.31	0.01	−0.65	−0.78	0.20	0.69	**1**								
Fried bean stew	−0.29	0.61	0.25	0.20	0.29	−0.13	−0.68	**1**							
Saltiness	−0.05	0.40	0.48	0.41	0.01	−0.23	−0.79	**0.96**	**1**						
Coarseness	0.74	**−0.89**	0.37	0.25	−0.77	−0.12	0.41	−0.73	−0.57	**1**					
Particulate	0.75	**−0.88**	0.41	0.27	−0.78	−0.11	0.39	−0.70	−0.54	**1.00**	**1**				
Beany after‐taste	**0.88**	−0.66	**0.99**	**0.96**	−0.79	−0.77	−0.66	0.19	0.41	0.39	0.41	**1**			
Burnt after‐taste	0.85	−0.63	**0.93**	**1.00**	−0.69	**−0.88**	−0.76	0.18	0.39	0.27	0.29	**0.96**	**1**		
Saltiness after‐taste	−0.38	0.67	0.11	0.15	0.36	−0.09	−0.72	**0.94**	**0.90**	−0.86	−0.84	0.07	0.12	**1**	
Viscosity	−0.73	0.68	−0.70	−0.43	0.82	0.15	−0.08	0.18	0.01	−0.79	−0.82	−0.65	−0.44	0.42	**1**

Note

Values in bold are significant at *p* ≤ .05.

### 3.2 Consumer preference

**FIGURE 2:**
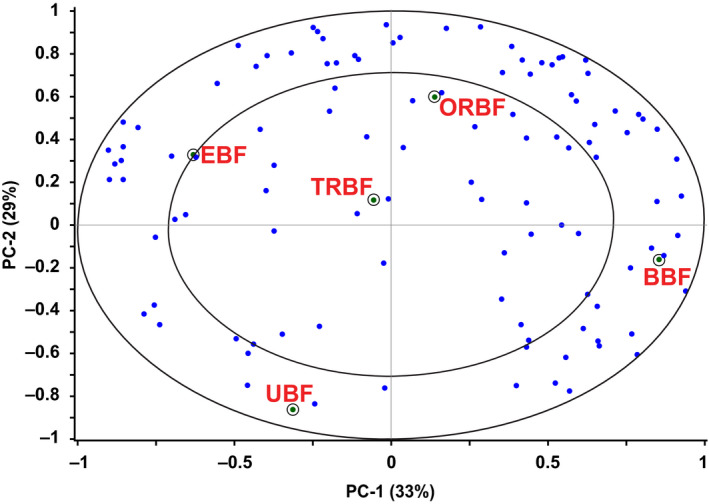
PCA results showing individual consumer preference of bean sauces. BBF, boiled bean flour; EBF, extruded bean flour; ORBF, oven roasted bean flour; TRBF, traditionally roasted bean flour; UBF, unprocessed bean flour

Internal preference mapping using PCA of the consumer preference scores showed that the first two PCs accounted for 62% of the total variation in the consumer preference data of the bean sauces (Figure 2). In the PCA, consumers were considered as the variables and bean sauces as the observations. There was a high concentration of consumers around BBF and ORBF indicating that a large group of consumers in the sample expressed their preference for these two types of sauces compared with the rest. It was also clear that more consumers expressed preference for sauce from EBF compared to those who did for sauces from TRBF and UBF. These results are contrary to those by Nkundabombi et al. (2016) who evaluated bean sauces from two common bean varieties (ROBA1 and K131) and reported that sauces from extruded and malted/roasted ROBA1 beans were equally liked whereas sauces from malted and roasted K131 beans had higher acceptability scores than those from extruded beans.

PC1 which explained 33% of the preference data variance, showed preference opposition between BBF (*r* = .86, PC1) and EBF (*r* = −.63, PC1). PC2 which explained an additional 29% of the preference data variance, also showed anti‐correlation between ORBF (*r* = .59, PC2) and sauce from UBF (*r* = −.87, PC2). This indicated that consumers who liked sauce from BBF did not like sauce from EBF and vice versa. This was the same scenario in the case of consumers who liked sauces from ORBF versus those who liked sauce from UBF.

Examining both PCA maps of the sensory, viscosity (Figure 1), and preference data (Figure 2) revealed that consumers liked the sauce from BBF probably because of its high expression of coarseness, particulate mouth‐feel, and millet porridge flavor and also because of its low fried bean stew flavor, saltiness, and saltiness after‐taste, and low viscosity. Those who liked the sauce from ORBF did so probably because of its being highly expressive of coarseness, particulate mouth‐feel, brown appearance, beany and burnt aromas, beany and burnt after‐tastes, and low viscosity. Lower preference seemed to be explained by lumpiness, mashed potato flavor, and higher viscosity in bean sauces which were all characteristic of UBF sauce. Internal preference mapping identified the general tendencies in preference of bean sauces, but it was clear that there was preference of some products over others due to possible consumer segmentation. An analysis using a segmentation technique that identified similarities among consumers was performed to further explain the pattern of preference.

### 3.3 Cluster analysis of the consumer preference data

Hierarchical cluster analysis showed that the sampled consumer population consisted of three clusters of consumers with homogenous preference patterns (Table 4). Mean preference scores obtained for the five bean sauces for all consumers were above average (5). Overall, consumers most preferred ORBF and least preferred UBF. However, examination of the preference scores within each of the three clusters revealed quite a different trend.

**TABLE 4:** Mean preference scores within each cluster for each of the bean sauces evaluated

Cluster size			Bean sauces				
Cluster	*n*	%	UBF	TRBF	BBF	EBF	ORBF
1	49	41	4.59^a^	5.78^a^	7.02^a,^ ^1^	4.57^a,^ ^2^	6.49^a^
2	31	26	4.55^a^	5.90^a,^ ^1^	3.39^b,^ ^2^	5.90^b,^ ^1^	5.61^b^
3	40	33	6.83^b^	6.70^b,^ ^2^	6.73^a^	7.28^c,^ ^1^	6.75^a^
*F* value	–		23.01	3.30	90.03	37.64	4.97
*p*‐value	–		.000	.040	.000	.000	.009
LSD	–		2.23	0.92	3.34	1.33	0.88
All^3^	120		5.33 (2.01)^2^	6.12 (1.81)	5.98 (1.98)	5.82 (1.86)	6.35 (1.61)^1^

Note

Means within a column with the same superscript (a–c) are not significantly different according to the Least Significant Difference (LSD) test results.

Abbreviations: BBF, boiled bean flour; EBF, extruded bean flour; ORBF, oven roasted bean flour; TRBF, traditionally roasted bean flour; UBF, unprocessed bean flour.

^1^ The most preferred sample within the cluster.

^2^ The least preferred sample within the cluster.

^3^ The Standard Deviation of the overall means of each sample is in parentheses.

The most preferred sauce was BBF in cluster 1, which was the largest cluster and, in this cluster, the least preferred sauce was EBF. In cluster 3 the second largest cluster, EBF was the most preferred and TRBF the least preferred sauce. In cluster 2 the smallest cluster, EBF and TRBF were the most preferred while BBF was the least preferred sauce.

**FIGURE 3:**
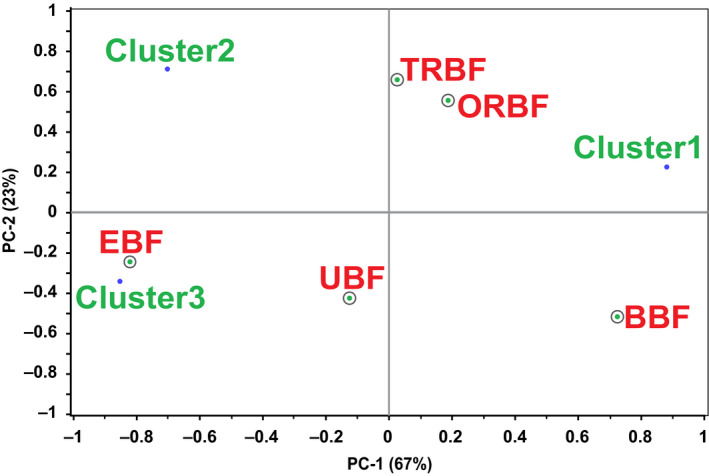
PCA results showing mean cluster consumer preferences of bean sauces. BBF, boiled bean flour; EBF, extruded bean flour; ORBF, oven roasted bean flour; TRBF, traditionally roasted bean flour; UBF, unprocessed bean flour

Principal component analysis results of mean preference scores of the clusters and the bean sauces showed that the first two PCs explained 90% of the preference data variation (Figure 3). PC1 which explained 67% of the variance in the preference data, predominantly described cluster 1 (*r* = .88, PC1) which was composed of consumers who most preferred sauce from BBF (*r* = .73, PC1). PC2, accounting for 23% of the variation in the preference data mainly described cluster 2 (*r* = .71, PC2) which had consumers whose most preferred sauce was from TRBF (*r* = .65, PC2). Cluster 2 (*r* = −.70, PC1) consumers also had EBF (*r* = −.82, PC1) as their most preferred sauce. Consumers in Cluster 3 (*r* = −.85, PC1) had EBF as their most preferred sauce too.

The earlier observed anti‐correlation between BBF and EBF was confirmed by the fact that in cluster 1 and 3, BBF and EBF featured interchangeably as the most and least preferred sauces respectively. EBF was the most preferred sauce in two out of the three clusters while ORBF did not feature in any of the clusters as the most preferred sample. Although ORBF was not the most preferred sample in any of the clusters, it was not the least preferred either in any of them.

According to the mean overall preference scores, the order of preference of the samples seemed to be, from most to least preferred, ORBF, TRBF, BBF, EBF, and UBF. The clustering preference suggested a different pattern. The largest cluster expressed BBF as the most preferred sample. Since BBF and EBF were antagonistic in consumer relationship, it could be assumed that EBF would seem to be the next more popular choice for the rest of the consumers especially since it featured in the remaining two clusters, particularly cluster 3 the second largest as the most preferred sauce. However, the mean preference score of cluster 3, in which EBF was dominant, had a low correlation (Figure 3) on PC2 which component described only 23% of the variation in the preference data in spite of cluster 3 being the second largest cluster. This implies that ORBF could have been the next most popular choice instead, for the reasons that consumers in cluster 1 had it as their second best (Table 4), it had a higher correlation than TRBF on PC1, which described the majority (67%) of the variation in the preference data and was closer to cluster 1 (Figure 3). In view of both cluster size and strength of the mean cluster preference scores, it could be suggested that the order of preference of the samples seemed to be, from most to least preferred, BBF, ORBF, TRBF, EBF, and lastly UBF.

### 3.4 Relationship between consumer preference and bean sauce descriptive sensory and viscosity data

Partial least squares regression analysis has wide applications in the food industry including detection of food authenticity, safety, and quality (Muhammad et al., 2020). It is also commonly used to investigate the sensory characteristics underlying consumer preference (Ares, Gimenez, & Gambaro, 2006; Tenenhaus, Pages, Ambroisine, & Guinot, 2005). PLSR has been used to appreciate differences between naturally and lactic acid bacteria‐fermented pastes of soybeans and soybean–maize blends (Ng'ong'ola‐Manani, Mwangwela, Schüller, Østlie, & Wicklund, 2014) and explain drivers of liking for snack products from extruded rice and pinto bean flours (Bernstein, 2015) and burgers made with micronized chickpea and green lentil flours (Shariati‐Ievari, 2013).

**FIGURE 4:**
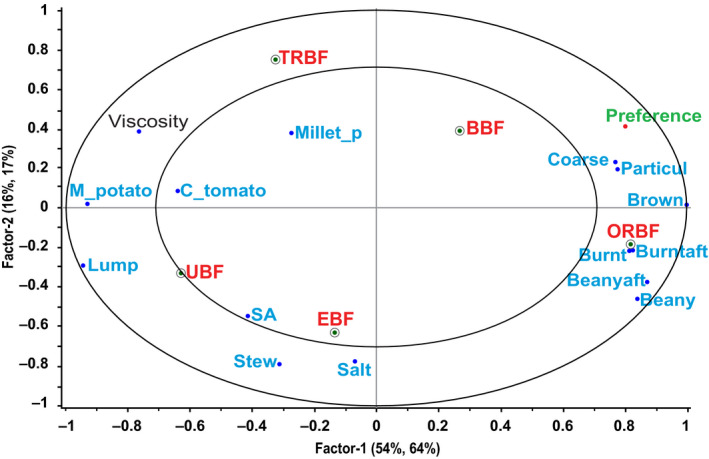
Partial least squares regression model 1 showing the relationship between sensory attributes, viscosity, and consumers' overall bean sauce preference score. BBF, boiled bean flour; Beanyaft, beany after‐taste; Burntaft, burnt after‐taste; C_tomato, cooked tomato; Coarse, coarseness; EBF, extruded bean flour; Lump, lumpiness; M_potato, mashed potato; Millet_p, millet porridge; ORBF, oven roasted bean flour; Particul, particulate; SA, salt after‐taste; Stew, fried bean stew; TRBF, traditionally roasted bean flour; UBF, unprocessed bean flour

The relationship between consumers' overall mean preference scores and bean sauce sensory characteristics and viscosity was investigated using a PLSR1 to identify the most relevant drivers of preference. The PLSR1 results showed that Factor 1 and Factor 2 together explained 70% of the variation in the bean sauces in terms of their sensory properties and viscosity, and 81% of the variation in their consumer preference. Brown color, burnt aroma, and burnt after‐taste were significantly highly correlated with preference (95% confidence interval did not contain zero value) and were therefore considered to be the most relevant sensory drivers of the bean sauce preference (Figure 4). Lumpiness and mashed potato flavor were significantly highly correlated with preference and since they loaded directly opposite the drivers of preference, they could be considered to be the drivers of dislike of bean sauces. The model correlation coefficient (*r* = .64) showed that the PLSR1 predicted the consumer preference fairly well.

As seen earlier, the preference order suggested from overall mean preference scores was in favor of sauce from ORBF as the highest ranked sauce. It is not unlikely therefore that descriptors typical of ORBF matched the most relevant sensory drivers identified for bean sauce preference. It should be noted however, that as Carbonell et al. (2008) reported, the use of overall mean values only captures the most manifest consumer preference patterns, whereas further categorizing major preference patterns can provide an opportunity to discover hidden information, masked by data averaging.

### 3.5 External preference mapping of the descriptive sensory attributes, viscosity, and cluster preference

**FIGURE 5:**
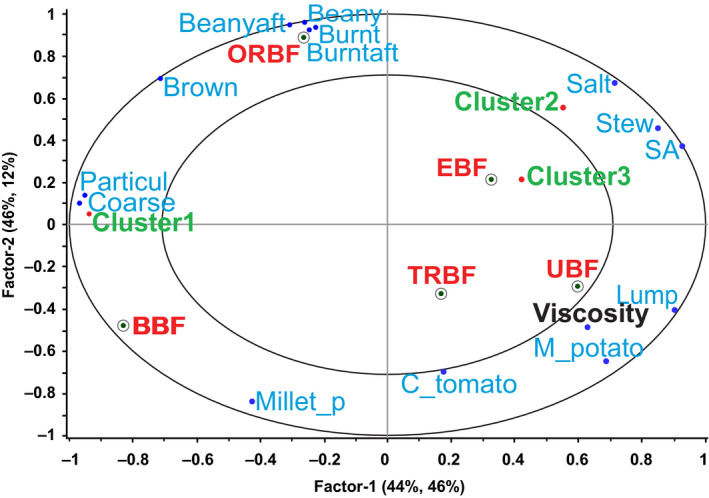
Partial least squares regression model 2 showing the external preference mapping of the bean sauces, their sensory attributes and viscosity, and consumer clusters. BBF, boiled bean flour; Beanyaft, beany after‐taste; Burntaft, burnt after‐taste; C_tomato, cooked tomato; Coarse, coarseness; EBF, extruded bean flour; Lump, lumpiness; M_potato, mashed potato; Millet_p, millet porridge; ORBF, oven roasted bean flour; Particul, particulate; SA, salt after‐taste; Stew, fried bean stew; TRBF, traditionally roasted bean flour; UBF, unprocessed bean flour

The relationship between consumer clusters' mean preference scores and bean sauce sensory characteristics and viscosity was investigated using a PLSR2 to identify the most relevant drivers of preference for each cluster. The results of the model showed that 90% of the sensory and viscosity data for Factor 1 and Factor 2 explained 58% of the clusters' bean sauce preferences (Figure 5). In Cluster 1, brown color and coarseness were significantly positively correlated with preference while lumpiness, fried bean stew flavor, and saltiness were significantly negatively correlated with preference (95% confidence interval did not contain zero value). In Cluster 2 and 3, none of the descriptors were significantly correlated with preference. Brown color and coarseness were therefore considered to be the most relevant sensory drivers of bean sauce preference in cluster 1 whereas lumpiness, fried bean stew flavor, and saltiness as the drivers of dislike. The ability of the model to fit the data was assessed by its correlation coefficient, which was high (*r* = .89).

## 4 CONCLUSIONS

Panelists clearly distinguished between the bean sauces more by their appearance, taste, and mouth‐feel than by the aroma, flavor, and after‐taste descriptors. Sauce from unprocessed flour had higher viscosity than sauces from the majority of the processed flours. Panelists were more able to differentiate sauces from ORBF, BBF, and UBF than those from TRBF and EBF. Lower preference seemed to be associated with lumpiness, mashed potato flavor, and higher viscosity in bean sauces. To the average consumer, a bean sauce with brown color, burnt aroma, and burnt after‐taste was the most preferred while one that was lumpy with high mashed potato flavor was the least preferred. ORBF was thus chosen over other sauces as the best. Among consumers in the largest cluster, brown color and coarseness of bean sauce were responsible for increased preference whereas lumpiness, fried bean stew flavor, and saltiness led to dislike. The majority of consumers chose sauce from BBF over other sauces as the best. At both overall and cluster level, preference was influenced positively by brown color and negatively by lumpiness. From these results, it can be concluded that appearance is a major driver of preference in bean sauces compared with other sensory attributes. Therefore, to increase utilization and acceptance of bean sauces and similar products from bean flour, product developers need to pay a lot of attention to appearance.

## CONFLICT OF INTEREST

The authors have no conflicts of interest.

## ETHICAL APPROVAL

This study does not involve any human or animal testing.

## INFORMED CONSENT

Written informed consent was obtained from all study participants.
